# Inter-arm blood pressure difference, when is it a useful risk marker for cardiovascular events?

**DOI:** 10.1038/s41371-021-00629-x

**Published:** 2021-11-06

**Authors:** Christopher E. Clark

**Affiliations:** Primary Care Research Group, Institute of Health Services Research, University of Exeter Medical School, College of Medicine & Health, Smeall Building, St Luke’s Campus, Magdalen Road, Exeter, Devon, EX1 2LU England

**Keywords:** Risk factors, Prognosis

Differences in the blood pressure between arms (inter-arm difference; IAD) are regularly encountered in assessing people with hypertension. A pooled analysis of community-based studies found systolic IADs ≥10 mmHg in 11.2% of people with hypertension, 7.4% with diabetes and 3.6% of a general adult population [1]. A systolic IAD ≥ 10 mmHg is associated with increased cardiovascular and all-cause mortality, and with cardiovascular events. We have recently confirmed this in an individual participant data (IPD) meta-analysis using 10 years of follow up data from over 50,000 participants pooled from 24 cohorts to establish the INTERPRESS-IPD Collaboration. We derived and validated prediction models which included systolic IAD as a risk marker, and confirmed the association of IAD with elevated risk after adjustment for frequently used cardiovascular risk prediction scores (ASCVD, Framingham and QRISK2) [2]. This work was designed to support interpretation of an IAD to stratify risk and inform management decisions within a primary care population.

In a linked paper Nolde et al. consider whether IAD could also be useful for the stratification of higher-risk groups. They examined the associations of IAD with vascular target organ damage in 199 patients recruited from a tertiary hypertension outpatient clinic; carotid-femoral pulse wave velocity (PWV) was adopted as a surrogate marker of hypertension-mediated vascular organ damage [3]. In this selected cohort, they were unable to show a cross-sectional relationship between IAD and PWV. Further analyses also failed to find an association of IAD with either absolute blood pressure (BP) levels or BP variability.

PWV provides a quantitative assessment of arterial stiffening and is a recognised risk marker for cardiovascular events [4]. The aetiology of an IAD is not clearly established; initially regarded as subclavian stenosis on the side of the lower reading arm, to date there has been a paucity of evidence to support such an association in the absence of substantial (i.e., ≥35 mmHg) systolic BP differences between arms [5]. There is a growing body of evidence to associate IAD with elevated arterial stiffness manifested as increased PWV and/or pulse pressure [6, 7]. It seems likely that IAD is largely, if not fully, mediated by asymmetries in arterial stiffness, and that this pathophysiology underlies the independent associations of IAD with cardiovascular risk. In truth, it is probable that both stenoses and stiffening of arteries make contributions to the phenomenon of IAD and since both pathologies share common underlying causes the distinction is, perhaps, somewhat academic [8]. We have previously observed that substantial sample sizes, probably in excess of 1000 for carotid-femoral PWV and higher for brachial-ankle PWV, are required before cross-sectional associations with an IAD can be reliably demonstrated [9]. Thus, the numbers examined by Nolde et al. may have been underpowered in their ability to demonstrate a true relationship, and restriction to dichotomous rather than continuous analysis may have further compounded this [10].

The cohort studied were drawn from a tertiary hospital hypertension clinic, whereas the INTERPRESS-IPD population included 56% with hypertension and selected clinical cohorts were not eligible for inclusion [2]. It is likely, therefore, that Nolde et al.’s failure to show the previously described association of magnitude of IAD with absolute BP could also be attributed to the restricted range of baseline BPs within their cohort [1–3, 11].

Whilst we have compelling data to show how detection of an IAD can be applied to refine risk assessment for primary prevention of events, thus informing individual treatment decisions, this association does not always hold true for people at higher risk. Magnitude of IAD has been correlated with severity of coronary artery disease as assessed by Gensini or SYNTAX scores in small studies (*n* = 104 and 106) of people presenting for angiography [12, 13]. In contrast, the much larger prospective SMART study of 7344 participants followed over a median of 5.9 years associated increasing systolic IAD with increased risks of vascular events in people without, but not with, pre-existing vascular disease after carefully adjusted analyses [14]. This highlights the point that cross-sectional associations of IAD with markers of cardiovascular risk cannot be assumed to translate directly into higher prospective risks of events. There will be degrees of residual confounding to account for; attendees for coronary arteriography or tertiary management of hypertension will already be classified as possessing high cardiovascular risk by any measure. Consequently, they should all be receiving interventions to lower BP and lipids without recourse to assessment of surrogate markers of risk. Risk stratification of higher-risk groups, whilst a perfectly valid research question, does not, therefore, carry the same potential to inform clinical management decisions as our findings for the INTERPRESS-IPD population [2].

Data held in the INTERPRESS-IPD Collaboration are largely derived from sequential blood pressure measurements [2]. As Nolde et al. correctly observe, the magnitude of such IAD measurements is greater than comparable simultaneous measurements [1, 3, 15]. In their study, they used the Microlife Watch BP Office device – a two cuff device capable of repeated simultaneous measures, although they only obtained a single pair of measurements for their study. This might account for the high (16.6%) prevalence of systolic IAD ≥ 10 mmHg that was found in this cohort, compared to our previously reported pooled prevalence of 11.2% (95% confidence interval [CI] = 9.1–13.6) in hypertension based on repeated simultaneous measurements from seven studies; repeated measures are associated with falling prevalences of IAD [1, 16]. Differences between IAD according to method of measurement can be attributed to common causes of BP variation such as order effects, white coat effects, and regression to the mean [16]. Such variation can be attenuated by controlled, repeated simultaneous measurement as is often adopted in research studies [11]. It is unfortunate that the opportunity for repeated measures offered by the Watch BP Office device was not taken, in order to improve accuracy of reported IADs. In considering sequential and simultaneous approaches, sequential BP measurement has a high negative predictive value for a simultaneous IAD, in other words, sequential measurement, despite its inherent inaccuracies, can reliably rule out an IAD where no significant IAD exists on simultaneous assessment [17]. Simultaneous measurement of BP in both arms requires the use of either two devices together, which will approximate to simultaneously measured data, or a single two cuff (or four limb) device. In primary care practitioners rarely have access to equipment that can measure both arms simultaneously, and they need a practical and simple method of assessment [18, 19]. Despite universal hypertension guideline advice to measure both arms when assessing people for hypertension, this at best appears to occur in about 50% of cases [19]. So, for practical reasons, an understanding of sequentially detected IADs is necessary since this is highly likely to be method of measurement adopted in primary care. Importantly, findings from both the INTERPRESS-IPD Collaboration and other sequentially measured cohorts consistently demonstrate the associations of IAD with all-cause mortality, cardiovascular mortality and cardiovascular events [2, 5, 20]. Thus experimental error in detection of IAD by sequential measurement may produce higher absolute IADs than repeated simultaneous assessment but is unlikely, in itself, to sufficiently explain associations with mortality and cardiovascular events.

In summary, Nolde et al.’s findings do not challenge the importance of including IAD in assessment of cardiovascular risk for primary prevention of future cardiovascular events and death. This paper does emphasise that selected higher-risk populations differ from those representative of the wider population. Risk stratification is arguably of less importance when all interventions that can reduce cardiovascular risk are already justified by pre-existing medical history. Importantly, the contrast in sample sizes is relevant and future studies of IAD can only be meaningful if they are adequately powered.

## References

[1] Clark C, Taylor R, Shore A, Campbell J (2016). Prevalence of systolic inter-arm differences in blood pressure varies for different primary care populations: systematic review and meta-analysis. Br J Gen Pr.

[2] Clark CE, Warren FC, Boddy K, McDonagh STJ, Moore SF, Goddard J, et al. Associations between systolic interarm differences in blood pressure and cardiovascular disease outcomes and mortality. Hypertension. 2021;77:650–61. 10.1161/HYPERTENSIONAHA.120.15997 PMC7803446 33342236

[3] Nolde JM, Lugo-Gavidia LM, Kannenkeril D, Chan J, Robinson S, Jose A, et al. Simultaneously measured inter-arm blood pressure difference is not associated with pulse wave velocity in a clinical dataset of at-risk hypertensive patients. J Hum Hypertens. 2021. 10.1038/s41371-021-00588-3. Epub ahead of print. 10.1038/s41371-021-00588-3 34354250

[4] Williams B, Mancia G, Spiering W, Agabiti Rosei E, Azizi M, Burnier M (2018). 2018 ESC/ESH guidelines for the management of arterial hypertension. Eur Heart J.

[5] Clark CE, Taylor RS, Shore AC, Ukoumunne OC, Campbell JL (2012). Association of a difference in systolic blood pressure between arms with vascular disease and mortality: a systematic review and meta-analysis. Lancet.

[6] Canepa M, Milaneschi Y, Ameri P, Alghatrif M, Leoncini G, Spallarossa P (2013). Relationship between inter-arm difference in systolic blood pressure and arterial stiffness in community-dwelling older adults. J Clin Hypertens.

[7] Iida M, Ishiguro Y, Ueda N, Honjo H (2020). Inter-arm difference of systolic blood pressure measured by automated double-cuff device is associated with arterial stiffness in patients with hypertension. Blood Press Monit.

[8] Clark C, Warren F, Boddy K, McDonagh S, Moore S, Cloutier L (2019). Inter-arm blood pressure difference: insights into aetiology from the INTERPRESS-IPD collaboration. J Hypertens.

[9] Clark CE (2017). The interarm blood pressure difference: do we know enough yet?. J Clin Hypertens.

[10] Altman DG, Royston P (2006). The cost of dichotomising continuous variables. BMJ.

[11] Omboni S, Verberk WJ (2019). Simultaneous double arm automated blood pressure measurement for the screening of subjects with potential vascular disease: a community study. Blood Press.

[12] Sadasivam K, Sundari M, Bhavsar NR, Ramraj B, Sampath A, Saravanan A. Relationship between inter-arm blood pressure difference (IAD) and severity of coronary artery disease. Ann Trop Med Public Health. 2020; 23.

[13] Durmuş G, Belen E, Bayyigit A, Kalyoncuoğlu M, Can MM (2018). The relationship between inter-arm blood pressure difference and coronary artery disease severity calculated by the SYNTAX score. Int J Hypertens.

[14] Kranenburg G, Spiering W, de Jong PA, Kappelle LJ, de Borst GJ, Cramer MJ (2017). Inter-arm systolic blood pressure differences, relations with future vascular events and mortality in patients with and without manifest vascular disease. Int J Cardiol.

[15] Verberk WJ, Kessels AGH, Thien T (2011). Blood pressure measurement method and inter-arm differences, a meta-analysis. Am J Hypertens.

[16] Schwartz CL, Clark C, Koshiaris C, Gill PS, Greenfield SM, Haque SM et al. Interarm difference in systolic blood pressure in different ethnic groups and relationship to the “white coat effect”: a cross-sectional study. Am J Hypertens. 2017;30:884–91. 10.1093/ajh/hpx073 PMC5861584 28475667

[17] Clark CE, Steele AM, Taylor RS, Shore AC, Ukoumunne OC, Campbell JL (2014). Inter-arm blood pressure difference in people with diabetes: measurement and vascular and mortality implications: a cohort study. Diabetes Care.

[18] Konya J, Mcdonagh S, Hayes P, Abel G, Boddy K, Clark C. Diagnosis of peripheral arterial disease in primary care: a survey of general practitioners in England & Ireland. In: Living & Dying Well: 49th Annual Scientific Meeting of the Society for Academic Primary Care. Leeds; Society for Academic Primary Care; 2021.

[19] Mejzner N, Clark CE, Smith LF, Campbell JL. Trends in the diagnosis and management of hypertension: repeated primary care survey in South West England. Brit. J. General Pract. 2017;67:e306–e313. 10.3399/bjgp17X690461 PMC5409425 28347984

[20] Weinberg I, Gona P, O’Donnell CJ, Jaff MR, Murabito JM (2014). The systolic blood pressure difference between arms and cardiovascular disease in the Framingham heart study. Am J Med.

